# Neuronal CC chemokines: the distinct roles of CCL21 and CCL2 in neuropathic pain

**DOI:** 10.3389/fncel.2014.00210

**Published:** 2014-08-07

**Authors:** Knut Biber, Erik Boddeke

**Affiliations:** ^1^Department of Psychiatry and Psychotherapy, University Hospital FreiburgFreiburg, Germany; ^2^Department of Neuroscience, University of Groningen, University Medical Center GroningenGroningen, Netherlands

**Keywords:** neuropathic pain, microglia reaction, chemokines, neuron-microglia signaling, DRG neurons, LDV vesicles, regulated release pathway

## Abstract

The development of neuropathic pain in response to peripheral nerve lesion for a large part depends on microglia located at the dorsal horn of the spinal cord. Thus the injured nerve initiates a response of microglia, which represents the start of a cascade of events that leads to neuropathic pain development. For long it remained obscure how a nerve injury in the periphery would initiate a microglia response in the dorsal horn of the spinal cord. Recently, two chemokines have been suggested as potential factors that mediate the communication between injured neurons and microglia namely CCL2 and CCL21. This assumption is based on the following findings. Both chemokines are not found in healthy neurons, but are expressed in response to neuronal injury. In injured dorsal root ganglion cells CCL2 and CCL21 are expressed in vesicles in the soma and transported through the axons of the dorsal root into the dorsal horn of the spinal cord. Finally, microglia *in vitro* are known to respond to CCL2 and CCL21. Whereas the microglial chemokine receptor involved in CCL21-induced neuropathic pain is not yet defined the situation concerning the receptors for CCL2 in microglia *in vivo* is even less clear. Recent results obtained in transgenic animals clearly show that microglia *in vivo* do not express CCR2 but that peripheral myeloid cells and neurons do. This suggests that CCL2 expressed by injured dorsal root neurons does not act as neuron-microglia signal in contrast to CCL21. Instead, CCL2 in the injured dorsal root ganglia (DRG) may act as autocrine or paracrine signal and may stimulate first or second order neurons in the pain cascade and/or attract CCR2-expressing peripheral monocytes/macrophages to the spinal cord.

## The importance of pain

An important aspect for the survival of all organisms is the sensation of potential harmful (noxious) threats, which often are experienced as pain (nociception). Accordingly, it has been known for a long time that, even humans with congenital insensitivity to pain often die as children because they fail to notice injuries and illnesses, which underlies the importance of proper nociception (see for review: Indo, 2001; Cox et al., 2006; Costigan et al., 2009). Nociceptive neurons, like all primary afferent neurons, innervate organs and the periphery. Their cell bodies are located in the dorsal root ganglia (DRG) meaning that these neurons reside outside of the central nervous system. There are two main types of nociceptive neurons, unmyelinated C fibers and thin myelinated Aδ fibers, that both mainly express so called transient receptor potential (TRP) channels in order to respond to intense mechanical or thermal stimuli (see for review: Dhaka et al., 2006; Szallasi et al., 2007). Nociceptive neurons project to the dorsal horn of the spinal cord (mainly to Lamina I and II) where they signal to second-order neurons that project to higher pain centers in hypothalamus and cortex. The nociceptive signal in the dorsal horn of the spinal cord is also transmitted to interneurons that are important for the fast nociceptive withdrawal reflex. The physiologic nociceptive signal occurs in response to acute stimuli and continues only in its presence; meaning that physiologically nociceptive pain is rather short lived.

## Inflammatory pain

When tissue damage is more severe and causing a subsequent inflammatory reaction, nociception is prolonged and sensitized, thus the pain sensing system of the injured body parts undergoes profound changes in its responsiveness (Scholz and Woolf, 2007; Latremoliere and Woolf, 2009; Ren and Dubner, 2010; Johnson et al., 2013). As a result of this pain hypersensitivity the affected body parts are protected from further physical contact, which is to aid the healing process. This type of pain or hypersensitivity is directly caused by local inflammation in the injured or infected body parts and is therefore called inflammatory pain. In fact one of the hallmarks of inflammation in general is pain.

There are several ways by which nociception is sensitized by inflammation. Inflammatory mediators might directly affect TRP channel activity. Several compounds of the “inflammatory soup” such as bradykinins, prostaglandins, leukotriene B4 and many others are known to sensitize TRPV1 activity (Szallasi et al., 2007). Furthermore, it is known that pro-inflammatory cytokines including IL-1β or TNFα also directly affect the signaling and excitability of sensory neurons (see for review: Uçeyler et al., 2009). Moreover, it has been shown that these pro-inflammatory cytokines induce the release of several neuropeptides, such as substance P (SP) or calcitonine gene-related peptide (CGRP) from C fibers, which in turn initiate a higher expression of pain sensing receptors and increased excitability in sensory neurons; a process called neurogenic inflammation (Uçeyler et al., 2009). Thus, the impact of inflammatory factors on the pain sensing system is manifold and yet by far not completely understood. The fact that injection of almost all known pro-inflammatory factors can cause temporary pain or pain hypersensitivity shows the robustness of this tight connection between inflammation and pain sensation. Being in aid of the healing process, inflammatory pain persists until the end of the repair process, it disappears when inflammation is over. Thus, although inflammatory pain may last for several weeks, it is generally temporary and thus reversible.

## The dark side of nociception: neuropathic pain

Physiological pain is generally connected to pathology and in aid of the organism. However, sometimes pain itself becomes the primary clinical problem, meaning that pathological pain neither protects nor supports healing. Pathological pain occurs when nociceptive thresholds are reduced such that normally innocuous stimuli become painful (allodynia) or when pain is sensed even in the absence of a given stimulus. These phenomena are called neuropathic pain and are due to changes higher up in the pain cascade (spinal cord or brain stem), which are summarized as central sensitization (Latremoliere and Woolf, 2009). Central sensitization is characterized by reduced inhibition and increased neuronal excitability/synaptic efficacy of the neurons of the nociceptive pathway, which as a result uncouples pain sensation from noxious stimuli (Latremoliere and Woolf, 2009).

Neuropathic pain is a consequence of damage of peripheral nerves possibly caused by mechanical trauma, metabolic disorders (diabetes), neurotoxic chemicals, infections or tumors (Dworkin et al., 2003). Neuropathic pain treatment has conventionally been applied on the basis of the underlying disease, which means that it was anticipated that treatment of the disease would resolve the pain symptoms (Dworkin et al., 2007). However, since the primary disease and the resulting peripheral nerve damage only initiates the cascade that subsequently leads to development and maintenance of neuropathic pain, such an etiological approach does not capture the essential feature of neuropathic pain; central sensitization. As a consequence potential treatments for neuropathic pain should prevent, inhibit or reverse the various mechanisms occurring in central sensitization (Latremoliere and Woolf, 2009).

Nerve damage surely causes an inflammatory reaction at the lesion site, which is why neuropathic pain shares many features with inflammatory pain. However, in contrast to inflammatory pain it is the nerve injury itself with its profound impact that most likely initiates central sensitization. For example, comparing the changes in gene expression in the DRG neurons in animals after induction of inflammatory pain (complete freund’s adjuvant (CFA) injection) or nerve injury (chronic constriction injury (CCI) model) revealed by far more changes in mRNA expression in the latter paradigm, where hundreds of genes (approximately 5% of all detected genes) were affected by the nerve injury (Costigan et al., 2002; Rodriguez Parkitna et al., 2006). These changes were probable due to the loss of trophic support from the target organ and/or caused by the various signals that are released at the site of injury. The most prominent changes in mRNA expression were attributed to the following functional classes: transcription and translation, cellular metabolism, cytoskeleton, neurotransmission and inflammation (Costigan et al., 2002). Those changes are most likely linked to survival and re-grow of the injured neurons, but also affect their sensitivity and signaling capacities.

## Central sensitization

The injured peripheral neurons with their cell bodies in the DRGs are not the only neurons of the pain axis that respond to nerve injury. Electrophysiological changes in second order neurons that project from lamina I and II of the dorsal horn to the brain are characteristic for central sensitization and thus important for the development of neuropathic pain. There is evidence that the down-regulation of the potassium-chloride transporter 2 (KCC2) in lamina I neurons, in response to peripheral nerve injury is leading to an alteration in the chloride equilibrium of those cells. This altered chloride equilibrium attenuates GABAergic inhibitory synaptic transmission, or may even switch GABAergic signals from inhibitory to excitatory (Coull et al., 2005). In lamina II, neurons cause peripheral nerve injury an increase in synaptic drive to excitatory neurons, whereas the opposite is the case for inhibitory neurons in lamina II (Biggs et al., 2010). Thus, peripheral nerve injury leads to a substantial state of disinhibition, due to loss of GABAergic inhibition and a reduction in glycinergic inhibitory signaling, which, in combination with a strengthened excitatory signaling is essential for neuropathic pain (Latremoliere and Woolf, 2009). These changes in dorsal horn neurons show that peripheral nerve damage is “recognized” in more central brain parts. Indeed various mRNA expression profiling experiments show that peripheral nerve injury not only affects the cell bodies of the injured nerve in the DRG (Costigan et al., 2002; Rodriguez Parkitna et al., 2006), but also leads to profound changes in the mRNA expression in the ipsilateral dorsal horn of the spinal cord (Griffin et al., 2007). Depending on the used peripheral nerve damage model these changes varied considerably, both qualitatively and quantitatively. After spared nerve injury (SNI) 184 mRNA transcripts were found changed in the spinal cord, 310 changes in the mRNA expression pattern were found in response to CCI and after spinal nerve ligation (SNL) 399 mRNA changes were observed (Griffin et al., 2007). All models have their own specific characteristics, which are for example reflected by the differences in the death rate of DRG neurons (see for review: Costigan et al., 2009) and may explain the differences in gene expression. However, all these different types of injury lead to neuropathic pain in animal models indicating that those 54 mRNAs that were shared by all three models might be important for central sensitization and neuropathic pain (Griffin et al., 2007). Interestingly, the largest functional group out of those 54 was associated with immune function (Griffin et al., 2007).

It has been recognized in the last decade that multiple immunological processes are participating in neuropathic pain phenomena. Peripheral nerve injury leads to an inflammatory reaction directly at the site of the injured nerve and of the DRGs, where an early and prominent infiltration of peripheral macrophages is found observed (see for review: Scholz and Woolf, 2007). Given the importance of central sensitization in neuropathic pain, however, it is required to understand the changes in the dorsal horn of the spinal cord. Here the situation with respect to peripheral macrophages is less clear. It was reported that an early and prominent infiltration by peripheral macrophages does not occur in the spinal cord; moreover, a depletion of peripheral macrophages did not affect the development of neuropathic pain (Rutkowski et al., 2000; Mitchell et al., 2008, ref 100 from Ren and Dubner). In agreement with these findings, it was shown that the blood-spinal cord barrier of the spinal cord is not greatly affected after spinal nerve injury (Abram et al., 2006; Lu et al., 2009; Calvo et al., 2010). On the other hand Zhang and co-workers described that, in response to peripheral nerve injury macrophages invade the spinal cord, where they subsequently differentiate into microglia-like cells (Zhang et al., 2007). Moreover, it was shown in another study that spinal nerve injury led to a rapid and transient opening of the blood-spinal cord barrier (Beggs et al., 2010). Thus, whether or not peripheral myeloid cells invade the spinal cord in response to peripheral nerve injury is an unresolved issue at the moment. Irrespective of these conflicting results it is widely believed that the first cellular reaction in response to peripheral nerve injury is a rapid change in microglia morphology and physiology (see for recent review: McMahon and Malcangio, 2009).

## Microglia

Microglia are the primary immune cells of the CNS parenchyma that are derived from mesoderm as they stem from very early myeloid cells (microglia precursors) that in the mouse at around embryonic day 8–9 invade the developing nervous tissue (see for review: Prinz and Mildner, 2011). Due to their origin microglia share many features with peripheral myeloid cells, but they also show brain specific properties (Ransohoff and Cardona, 2010; Prinz and Mildner, 2011). In the adult brain and spinal cord microglia are more or less evenly distributed, and it is undisputed that these cells are the first line of defence which are activated upon any type of brain injury (Kreutzberg, 1996; Streit, 2002; van Rossum and Hanisch, 2004; Hanisch and Kettenmann, 2007; Biber et al., 2006). Microglia have small cell bodies, fine, long and heavily branched (ramified) processes that claim a territory which does not overlap with the territory of neighboring microglia. Life cell imaging studies using two-photon microscopy have shown that microglia rapidly move those processes in the non-challenged brain thereby palpating their direct environment, making them very active “surveillant” cells, rather than “resting” as long been thought (Nimmerjahn et al., 2005; Ransohoff and Cardona, 2010). In line with this “surveillance” function it was observed that microglia respond to cell damage rapidly within several minutes (Nimmerjahn et al., 2005) with changes in their morphology that follow a stereotypic pattern (Kreutzberg, 1996; Streit, 2002). Since these morphological changes are stereotypic and occur irrespective of the type of insult, the term “activated microglia” became misleading over the years, because it suggests a single functional state of those cells, which is known now not to be true (Hanisch and Kettenmann, 2007; Ransohoff and Cardona, 2010). It is now clear that microglia respond with a variety of different reactions by integrating multifarious inputs (Schwartz et al., 2006; Biber et al., 2007; Hanisch and Kettenmann, 2007; Ransohoff and Perry, 2009; Ransohoff and Cardona, 2010). It is therefore concluded that general terms like “microglia activation” or “activated microglia” are not sufficient to depict the function of microglia. Instead the different functional states of microglia should be described with respect to a given physiological or pathological situation (McMahon and Malcangio, 2009; Biber et al., 2014).

## Microglia in neuropathic pain

Approximately two decades ago it was recognized that dorsal horn microglia respond to peripheral nerve injury with a morphological change and up-regulation of several microglial markers (Eriksson et al., 1993). These findings, together with early observations that inflammatory mediators are involved in neuropathic pain (Watkins et al., 1994, 1995; DeLeo et al., 1997) and the discovery that the microglial reaction in the spinal cord and the development of neuropathic pain timely coincide (Colburn et al., 1997, 1999; Coyle, 1998) have raised the assumption that microglia are involved in neuropathic pain development (Watkins et al., 2001). It is clear today that inhibition of various microglia-specific receptors or effector molecules prevents the development of neuropathic pain (Jin et al., 2003; Schäfers et al., 2003; Tsuda et al., 2003; Terayama et al., 2008; Clark et al., 2009, 2010). Taken together, it is widely accepted that microglia function is crucial for the initiation of neuropathic pain (see for review: Ji et al., 2006; McMahon and Malcangio, 2009; Svensson and Brodin, 2010; Trang et al., 2012; Clark et al., 2013; Tsuda et al., 2013). However, while much has been revealed about the function of numerous microglia factors and receptors like P2X4, P2X7, TLR2, CX3CR1, BDNF and CatS (see fore excellent and recent reviews: Ji et al., 2006; McMahon and Malcangio, 2009; Svensson and Brodin, 2010; Trang et al., 2012; Clark et al., 2013; Tsuda et al., 2013) comparably little is yet know about the mechanisms that initiate the microglia response after peripheral nerve injury. From a therapeutically point of view, however, it would be of crucial interest to identify the signals that turn on the microglia response after peripheral nerve injury.

## Chemokines: effective signaling molecules in the brain

The CNS is spatially highly organized. In general neuron-neuron communication in the CNS is based on the regulated release of various signaling molecules, like neurotransmitters, neuropeptides, neurohormones and neurotrophins. With few exceptions, the release of these signaling molecules occurs at specific sites, for example synapses between neurons. This specific release requires a targeted intracellular transport of signaling molecules to these sites. Accordingly, neurons have various systems for the sorting, transportation and release of their numerous signaling molecules. Neurotransmitters are generally found in small, so-called synaptic vesicles, which undergo recycling and are loaded with neurotransmitters at the synapses. All protein or peptide signaling molecules are delivered to the membrane in either the constitutive or the regulated release pathway. This protein cargo is synthesized in the endoplasmatic reticulum (ER) and sorted in the trans-golgi-network (TGN) of the neurons. The vesicles of the regulated release pathway belong to the large dense core vesicles (LDV), with which neurons are able to sort, transport and release protein-signaling molecules like neurotrophins or neuropeptides at distinct sub-cellular sites (see for review: van Vliet et al., 2003; Salio et al., 2006; Gottmann et al., 2009; Zhang et al., 2010). Synapses between neurons are no longer considered the only communication points in the CNS since there is accumulating evidence for extrasynaptic release of signaling molecules and since there is considerable communication ongoing also between neurons and surrounding glia cells (Biber et al., 2007; Araque and Navarrete, 2010; Faissner et al., 2010; Giaume et al., 2010). Thus the concept of intracellular communication in the CNS has substantially broadened and therefore it is not surprising that new families of molecules are discussed at the moment to be messengers in the brain.

Chemokines are small proteins (10–20 kDa) and originally known from the peripheral immune system, where they orchestrate various aspects of immunity. Originally chemokines were described as chemotaxis-inducing cytokines; however, today it is clear that chemokines control numerous aspects of immune function making them important signaling molecules in health and disease (Borroni et al., 2010; Sharma, 2010). The first reports on chemokine expression in the brain focused on glia cells and their potential role in neuroimmunology (Biber et al., 2002). Apart from their expression in glia cells, at least five different chemokines (CCL2, CCL21, CXCL10, CXCL12 and CX3CL1) have been described in neurons in the last few years, predominately under conditions of neuronal stress or injury (de Haas et al., 2007; Biber et al., 2008; Miller et al., 2008). Since these chemokines have electrophysiological effects in neurons (Oh et al., 2002; Callewaere et al., 2006; Guyon et al., 2009; Miller et al., 2009) and control glia cell function in brain pathology (Cardona et al., 2008; Ransohoff, 2009), an important function of these neuronal chemokines in conveying signals from injured neurons has been suggested (de Haas et al., 2007; Ransohoff, 2009). The role of chemokines as microglia instruction signals has gained particular interest in the field of neuropathic pain, where at least three different neuronal chemokines (CX3XL1, CCL2 and CCL21) are playing different roles. Since the contribution of CX3CL1/CX3CR1 signaling in neuropathic pain is covered by Clark and Malcangio in this special research topic in Frontiers in Cellular Neuroscience (Clark and Malcangio, 2014), we here will focus on CCL2 and CCL21.

## Neuronal CCL2 and CCL21 and their potential role in neuropathic pain

The chemokines CCL2 and CCL21 have both been described to be up-regulated in injured DRG neurons (Zhang et al., 2007; Jung et al., 2009; Miller et al., 2009; Biber et al., 2011) and their role as neuron-microglia signaling factors involved in development of neuropathic pain has been proposed (Zhang et al., 2007; Jung et al., 2009; Miller et al., 2009; Biber et al., 2011). Both CCL2 and CCL21 are induced in the cell bodies of DRG neurons that are located outside of the spinal cord. There would be thus two prerequisites for effective microglia activation by neuronal chemokines in the spinal cord: first adequate transport of these chemokines from the DRG into the spinal cord is required and second spinal microglia should express of the corresponding receptors for CCL2 and CCL21.

## Sorting and transport of neuronal CCL21 and CCL2

The first evidence that CCL21 is specifically expressed in endangered neurons and may act as a signal from damaged neurons to microglia was published more than a decade ago (Biber et al., 2001). In subsequent studies in mice with disturbed CCL21 signaling inhibited microglia responses at the projection site of injured neurons were found and it was speculated that CCL21 is transported to axon endings (Rappert et al., 2004; de Jong et al., 2005). Corroborating this assumption it was observed that neuronal CCL21 is located in vesicles in neuronal cell bodies, axons and pre-synaptic terminals (de Jong et al., 2005). Subsequently CCL21-containing vesicles were identified as LDVs and their preferential transport towards the axon ends was shown (de Jong et al., 2008). These data were recently confirmed in dorsal root ganglion cells, in which CCL21 expression is induced by mechanical injury with subsequent transport of CCL21 through the dorsal root into the primary afferents in the spinal cord (Biber et al., 2011).

Similarly there is solid evidence from various models of neuropathic pain that CCL2 is strongly upregulated in DRG neurons (Tanaka et al., 2004; White et al., 2005; Zhang and De Koninck, 2006; Yang et al., 2007; Jung et al., 2008, 2009; Bhangoo et al., 2009; Jeon et al., 2009; Thacker et al., 2009; Van Steenwinckel et al., 2011). There is however, conflicting evidence about the transport of CCL2 from the DRG into the dorsal horn of the spinal cord. Whereas immunohistochemical findings suggested the transport of CCL2 from the DRG into the spinal cord (Zhang and De Koninck, 2006; Thacker et al., 2009; Van Steenwinckel et al., 2011), a report on CCL2-mRFP1 expressing transgenic mice showed that CCL2 expression was restricted to the lesioned DRG (Jung et al., 2009). Since different lesion models of the spinal nerve were used in these studies the question whether or not CCL2 is transported from the DRG to the spinal cord might depend on the lesion model.

The transport of CCL2, however, would require that CCL2 (like CCL21) is sorted into vesicles that allow such transport. Indeed, there also is evidence that CCL2 is expressed in neuronal vesicles (Jung et al., 2009) and a recent report using electron microscopy described CCL2 expression in small clear vesicles and LDV (Van Steenwinckel et al., 2011) suggesting that like CCL21 also CCL2 is sorted into vesicles of the regulated release pathway which would allow its directed transport and release. However, the mechanism of how neuronal chemokines are being sorted into LDV is a yet not explored question.

The classic cargo of LDV like neurohormones, neuropeptides and neurotrophins are all synthesized in a pre-pro-form and sorted in the TGN (see for review: van Vliet et al., 2003; Salio et al., 2006; Gottmann et al., 2009; Zhang et al., 2010). The “pre” of the pre-pro-form indicates the N-terminal signal peptide which is cleaved to allow the entry of the protein into the ER (van Vliet et al., 2003). Such N-terminal signal was also described for CCL21 and its deletion resulted in cytoplasmic expression of the chemokine showing that the entry into the ER is essential for the sorting of CCL21 (de Jong et al., 2008). Interestingly, bioinformatically methods using the online software SignalP3.0^1^ would propose such N-terminal signal also for CCL2, which would be cleaved off between position 23 and 24. Whether or not the deletion of this proposed N-terminal signal would also result in cytoplasmic expression of CCL2 is currently not known. However, the entry into the ER only is the first step of the sorting procedure and also is required for cargo that is sorted into the constitutive release pathway (see for review: van Vliet et al., 2003; Salio et al., 2006; Gottmann et al., 2009; Zhang et al., 2010). For the further sorting of cargo of the regulated release pathway into LDVs various proteases are involved and there is convincing evidence that the processing of the pro-form is required for the differential sorting of the cargo. Accordingly, various molecular sorting signals in the pro-form of LDV cargo have been identified (see for review: van Vliet et al., 2003; Salio et al., 2006; Gottmann et al., 2009; Zhang et al., 2010).

In contrast to classical LDV cargo, neuronal chemokines are not synthesized in a pre-pro-form, but in a pre-form, meaning that they only have the N-terminal signal peptide allowing them to enter the ER. Therefore, it is currently not understood how exactly CCL21 and potentially CCL2 in neurons are subjected to specific sorting into LDVs. However, the fact that both CCL21 and most likely CCL2 are sorted into LDVs the possibility arises the possibility that both chemokines are transported to different locations in neurons.

Taken together, various lines of evidence show that nerve injury causes the expression of the chemokines CCL2 and CCL21 in peripheral neurons. After injury, their rapid expression first is detected in the cell bodies of the neurons lying peripherally in the DRG, after which both chemokines are most likely transported through the dorsal root into the primary afferents in the spinal cord. Thus both chemokines fulfil the first requirement of being a signal that conveys the message of nerve damage from the periphery into the spinal cord.

It is interesting to note here that CCL21 has yet never been detected in healthy neurons, glia cells or other non-neuronal cells in the brain such as endothelial cells. Thus, CCL21 in the CNS is exclusively expressed in injured neurons and thus is one the few inflammatory mediators in the CNS with such exclusive cell specificity indicating a special role of this chemokine for the communication between injured neurons and their surroundings. In contrast, next to its neuronal expression, CCL2 in the brain has been additionally described in glia cells (astrocytes, microglia) (Biber et al., 2002). Furthermore, in peripheral nerve injury and development of neuropathic pain expression of CCL2 has been described in other cells than the injured DRG neurons, indicating that being a potential message to microglia most likely is not the only function of CCL2 after peripheral nerve injury (see below).

### CCR2: a chemokine receptor in microglia?

Since microglia are of myeloid origin and share many properties with peripheral monocytes/macrophages it was expected that microglia express the receptor for CCL2, formerly called monocyte chemoattractant protein-1 (MCP-1). There are thus various reports in which CCR2 expressing cells are suggested to be microglia (Abbadie et al., 2003; Zhang et al., 2007; Fernández-López et al., 2012) or described as microglia/macrophages (Yao and Tsirka, 2012) or referred to as amoeboid microglia cells (Deng et al., 2009). Often CCR2 is discussed to be an important receptor for the recruitment of microglia to injured brain areas (El Khoury et al., 2007; Zhang et al., 2007; Deng et al., 2009; Raber et al., 2013) and in this respect CCR2 has been described as receptor in spinal cord microglia that enables these cells to respond to peripheral nerve injury (Abbadie et al., 2003; Zhang et al., 2007).

On the other hand there is convincing evidence that microglia do *not* express CCR2. Various recent mRNA expression studies in acutely isolated microglia from the adult mouse brain did not detect CCR2 mRNA expression in these cells (Olah et al., 2012; Beutner et al., 2013; Hickman et al., 2013; Butovsky et al., 2014) nor was CCR2 mRNA expression earlier found in cultured microglia (Zuurman et al., 2003). Two different studies using transgenic mouse models in which CCR2-expressing cells were fluorescently labelled failed to detect the corresponding fluorescent signal in microglia in the healthy brain and in various disease models such as experimental autoimmune encephalomyelitis (EAE), LPS-injection and sciatic nerve demyelination (Jung et al., 2009; Mizutani et al., 2012). Finally there are various bone-marrow transplantation studies and experiments with parabiotic mice that show CCR2 expression solely in peripheral monocytes/macrophages that have invaded the diseased central nervous system (Mildner et al., 2007; Schilling et al., 2009a,b; Prinz and Mildner, 2011; Mizutani et al., 2012).

How is this controversy around CCR2 expression in microglia explained? With respect to their origin it is clear now that microglia are derived from primitive c-kit+ erythromyeloid yolk sac precursor cells that appear as early as embryonic day 8 in the mouse (Ginhoux et al., 2010; Kierdorf et al., 2013). Importantly, only these cells invade the developing nervous tissue and mature into microglia. Microglia never exchange with cells that stem from fetal liver- or bone-marrow haematopoiesis, making microglia a myeloid cell population in the adult that is exclusively derived from primitive haematopoiesis (Ginhoux et al., 2010; Schulz et al., 2012; Kierdorf et al., 2013). Microglia therefore are a specialized and local cell population, that most likely display self-renewing capacities without exchange with peripheral cells under physiological conditions (Ajami et al., 2007; Ginhoux et al., 2013). Since CCR2+/Lys6C high inflammatory monocytes, the cells that may enter the diseased brain, are derived from definitive haematopoiesis they are of different origin as microglia, yet it is extremely difficult to distinguish both populations in the diseased brain (see for recent review: Ginhoux et al., 2013; Neumann and Wekerle, 2013; Biber et al., 2014). Since it was shown that peripheral nerve injury led to a rapid (within 24 h) and transient (up to 7 days) opening of the blood-spinal cord barrier (Beggs et al., 2010) and that CCR2-postive peripheral cells enter the spinal cord in response to peripheral nerve injury (Zhang et al., 2007), the controversy about CCR2 expression in spinal cord microglia could potentially be due to CCR2+ inflammatory monocytes that have entered the spinal cord where they have been mistaken for endogenous microglia.

The lack of CCR2 in microglia would not support a role for neuronal CCL2 as microglia signal, however, the importance of CCL2 and its receptor CCR2 for the development of nerve-injury induced neuropathic pain is undisputed. There is an overwhelming body of literature that interfering with the CCL2-CCR2 system (antagonists, knockouts, inhibitor studies) reduces or prevents the development of neuropathic pain (see for recent reviews: Gao and Ji, 2010; Clark et al., 2013). It is obvious that the role of CCL2-CCR2 in this pathological pain state is mnifold and likely acts on various levels. Given the known role of CCL2 as an attracting factor for peripheral myeloid cells in the CNS it is most likely that CCL2 also in the spinal cord is important for the infiltration with monocytes/macrophages (Zhang et al., 2007). However, CCR2 is not only expressed in peripheral myeloid cells but also in DRG neurons and potentially in second order neurons in lamina II of the spinal cord (Gao et al., 2009; Jung et al., 2009). In these neurons several pro-nociceptive electrophysiological effects of CCL2 like enhancement of enhance glutamate receptor function or reduction of GABAergic signaling (Gosselin et al., 2005; Gao et al., 2009; Gao and Ji, 2010; Clark et al., 2013). Thus CCL2 in the DRG may act as autocrine signal (neuron-neuron signal) and paracrine in the spinal cord where neuronally released CCL2 may stimulate second order neurons in the pain cascade. The primary afferents of the DRG neurons are, however not the only cellular source of CCL2, as also spinal cord astrocytes express CCL2 under conditions of neuropathic pain (Gao and Ji, 2010; Clark et al., 2013). Thus interfering with CCL2 signaling may inhibit neuropathic pain development at various levels. Since microglia responses and neuropathic pain development are closely connected to each other, it may very well be that an inhibition of the pain cascade (by CCL2 antagonists for example) also inhibits the pain-related reaction of microglia. Such findings, however, are no formal proof of a direct effect of CCL2 in microglia.

### CCL21 receptors in microglia

Using CCL21-deficient mice (plt mutation) an important role of this neuronal chemokine in the development of neuropathic pain was demonstrated. Without neuronal CCL21 expression, animals did not develop signs of tactile allodynia in response to spinal nerve injury (Biber et al., 2011). This lack of neuropathic pain was due to a failure in microglia to up-regulate P2X4 expression after spinal nerve injury (Biber et al., 2011). In cultured microglia P2X4 mRNA and protein was induced by CCL21 stimulation showing that this chemokine is the responsible neuronal trigger for P2X4 up-regulation in microglia and the development of neuropathic pain (Biber et al., 2011), raising the question which microglia receptor is responsible here.

There are two known receptors for CCL21 in mice: CCR7 and CXCR3 (Biber et al., 2006). The main receptor for CCL21 is CCR7, which is not found in microglia under basal conditions, but it can be induced *in vitro* and *in vivo* (Biber et al., 2001, 2002; Rappert et al., 2002; Dijkstra et al., 2006). In contrast, CXCR3 is constitutively expressed in cultured microglia and in acutely isolated microglia (Biber et al., 2001, 2002; Rappert et al., 2002; de Haas et al., 2008). Thus cultured non-challenged microglia from CXCR3-deficient animals are not responsive to CCL21 stimulation (Rappert et al., 2002) but gain reactivity to CCL21 after immunological challenges (Dijkstra et al., 2006). Furthermore, CXCR3-deficient animals display markedly reduced microglia activation after neuronal injury in the entorhinal cortex lesion model (Rappert et al., 2004), indicating a prominent role of CXCR3 in microglia for the detection of neuronal damage in the nervous system. In order to understand which CCL21 receptor is involved in the development of neuropathic pain, CCR7-/- and CXCR3-/- animals were subjected to peripheral nerve damage. CCR7-deficient animals displayed a somewhat milder disease course, especially during the first days after spinal nerve injury (Biber et al., 2011). This delay in allodynia development might point to an induction of CCR7 expression in activated dorsal horn microglia, similar to what was found in a mouse model of multiple sclerosis (Dijkstra et al., 2006). However, in agreement with earlier studies we were not able to detect any CCR7 mRNA in the control spinal cord, neither was CCR7 mRNA induced by the nerve lesion. Given this lack of CCR7 in spinal cord tissue, the slightly milder disease development after spinal nerve injury in CCR7-deficient animals is most likely due to a yet not understood effect in the periphery. Surprisingly, the development of neuropathic pain was also not affected in CXCR3-deficient animals (Biber et al., 2011). Thus neither the deficiency of CCR7 or CXCR3 had a profound impact on the development of neuropathic pain, in contrast to the striking phenotype in the absence of their ligand CCL21.

The fact that only CCL21, but not the specific CXCR3 ligand CXCL10 or the specific CCR7 ligand CCL19 were able to induce P2X4 mRNA expression in cultured mouse microglia might point to another CCL21 receptor in these cells. Indeed, we have recently provided functional evidence for a third, yet not identified, CCL21 receptor in mouse glia cells (van Weering et al., 2010), indicating that the question of CCL21 receptors in glia cells is more complex than originally anticipated. Taken together, the responsible receptor for the CCL21-dependent development of neuropathic pain after spinal nerve injury remains to be established.

## Conclusions

**Figure 1:**
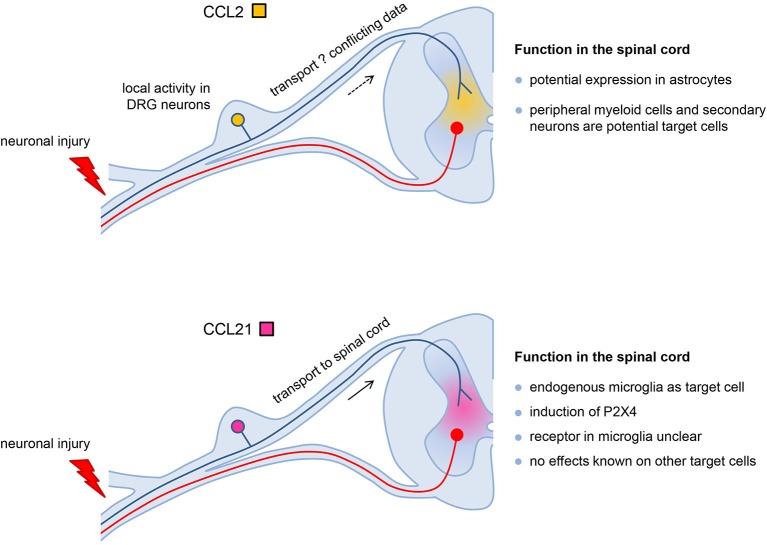
**The different roles of CCL2 and CCL21 in the development of neuropathic pain**. Both chemokines are induced in DRG neurons in response to nerve injury. CCL2 in the injured DRG may act as local autocrine signal (neuron-neuron signal) and potentially paracrine in the spinal cord where neuronally released CCL2 may stimulate second order neurons in the pain cascade and/or attract CCR2-expressing peripheral monocytes/macrophages. Since there are conflicting data about the transport of CCL2 from the DRG into the spinal cord, alternatively CCL2 from astrocytes might also activate these target cells. Neuronal CCL21 is transported from the DRG into the spinal cord and contributes to neuron-microglia signaling. CCL21 is the crucial trigger to up-regulate P2X4 receptors in spinal cord microglia which is a vital step in the cascade that leads to neuropathic pain. Although the receptor for CCL21 in spinal cord microglia is an unsolved issue, this chemokine most likely acts as neuron-microglia signal only, since effects of CCL21 in other cells of the spinal cord have yet not been described.

Despite the similar expression pattern in response to peripheral nerve injury there are clear differences in function of neuronal CCL2 and CCL21 in the development of neuropathic pain (Figure 1). CCL2 in the injured DRG may act as local autocrine signal (neuron-neuron signal) and paracrine in the spinal cord where neuronally released CCL2 may stimulate second order neurons in the pain cascade and/or attract CCR2-expressing peripheral monocytes/macrophages. Neuronal CCL21 contributes to neuron-microglia signaling and is the crucial trigger to up-regulate P2X4 receptors in spinal cord microglia, a vital step in the cascade that leads to neuropathic pain. Thus both neuronal chemokines play important roles in neuropathic pain development are potential drug targets to prevent the formation of neuropathic pain in response to peripheral nerve injury.

## Conflict of interest statement

The authors declare that the research was conducted in the absence of any commercial or financial relationships that could be construed as a potential conflict of interest.

## Footnotes

^1^http://www.cbs.dtu.dk/services/SignalP/
